# Molecular dynamics provides new insights into the mechanism of calcium signal transduction and interdomain interactions in cardiac troponin

**DOI:** 10.1002/2211-5463.13009

**Published:** 2021-06-09

**Authors:** Georgi Z. Genchev, Minae Kobayashi, Tomoyoshi Kobayashi, Hui Lu

**Affiliations:** ^1^ Center for Biomedical Informatics Shanghai Children's Hospital Shanghai China; ^2^ SJTU‐Yale Joint Center for Biostatistics Shanghai Jiao Tong University Shanghai China; ^3^ Bulgarian Institute for Genomics and Precision Medicine Sofia Bulgaria; ^4^ Bioinformatics Program, Department of Bioengineering University of Illinois at Chicago Chicago IL USA; ^5^ Department of Physiology and Biophysics and Center for Cardiovascular Research University of Illinois at Chicago College of Medicine Chicago IL USA; ^6^ Department of Bioinformatics and Biostatistics Shanghai Jiao Tong University Shanghai China

**Keywords:** calcium, calcium sensitizers, cardiac troponin, excitation–contraction coupling, molecular dynamics, skeletal troponin

## Abstract

Understanding the regulation of cardiac muscle contraction at a molecular level is crucial for the development of therapeutics for heart conditions. Despite the availability of atomic structures of the protein components of cardiac muscle thin filaments, detailed insights into their dynamics and response to calcium are yet to be fully depicted. In this study, we used molecular dynamics simulations of the core domains of the cardiac muscle protein troponin to characterize the equilibrium dynamics of its calcium‐bound and calcium‐free forms, with a focus on elements of cardiac muscle contraction activation and deactivation, that is, calcium binding to the cardiac troponin Ca^2+^‐binding subunit (TnC) and the release of the switch region of the troponin inhibitory subunit (TnI) from TnC. The process of calcium binding to the TnC binding site is described as a three‐step process commencing with calcium capture by the binding site residues, followed by cooperative residue interplay bringing the calcium ion to the binding site, and finally, calcium–water exchange. Furthermore, we uncovered a set of TnC–TnI interdomain interactions that are critical for TnC N‐lobe hydrophobic pocket dynamics. Absence of these interactions allows the closure of the TnC N‐lobe hydrophobic pocket while the TnI switch region remains expelled, whereas if the interactions are maintained, the hydrophobic pocket remains open. Modification of these interactions may fine‐tune the ability of the TnC N‐lobe hydrophobic pocket to close or remain open, modulate cardiac contractility and present potential therapy‐relevant targets.

## Graphical abstract

Cardiac muscle contraction involves a plethora of molecular actors. In cardiomyocytes, the sensor molecule troponin responds by a conformational change to a signal, calcium binding, which triggers the next steps leading to muscle contraction. This work explores the structural details of calcium binding, structural rearrangements and key interactions within this molecular sensor system.

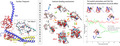

**Abbreviations**
CD: Circular dichroism
fs: fast skeletal
MD: molecular dynamics
N‐lobe: N‐terminal lobe
NMR: Nuclear Magnetic Resonance
PDB: Protein Data Bank
PKA: protein kinase A
SMD: steered molecular dynamics
TnC: troponin Ca^2+^‐binding subunit
TnI: troponin inhibitory subunit
TnT: troponin tropomyosin‐binding subunit
vdW: van der Waals

Cardiac troponin (a sarcomere protein) plays an important role in the Ca^2+^‐dependent regulation of cardiac contractility [1]. Cardiac muscle contraction is the result of the sliding between the thick and thin filaments; the thick filaments are mainly composed of myosin, and troponin, together with tropomyosin and actin, forms the thin filaments in striated muscle. Cardiac troponin is composed of three subunits: the Ca^2+^‐binding subunit (TnC), the inhibitory subunit (TnI) and the tropomyosin‐binding subunit (TnT). TnC is a dumbbell‐shaped E‐F hand‐type Ca^2+^‐binding protein (Fig. 1A). The Ca^2+^‐binding site(s) in the N‐terminal lobe (N‐lobe) of TnC has (have) a lower affinity for Ca^2+^ and is (are) considered to be responsible for the regulation process of muscle contraction [2, 3]. The targeted and precise interdomain interactions, which form the basis of the regulatory conformational response to the rapid fluctuation of Ca^2+^ levels in cardiomyocytes, are key determinants of keeping the function of the heart pump.

**Fig. 1:**
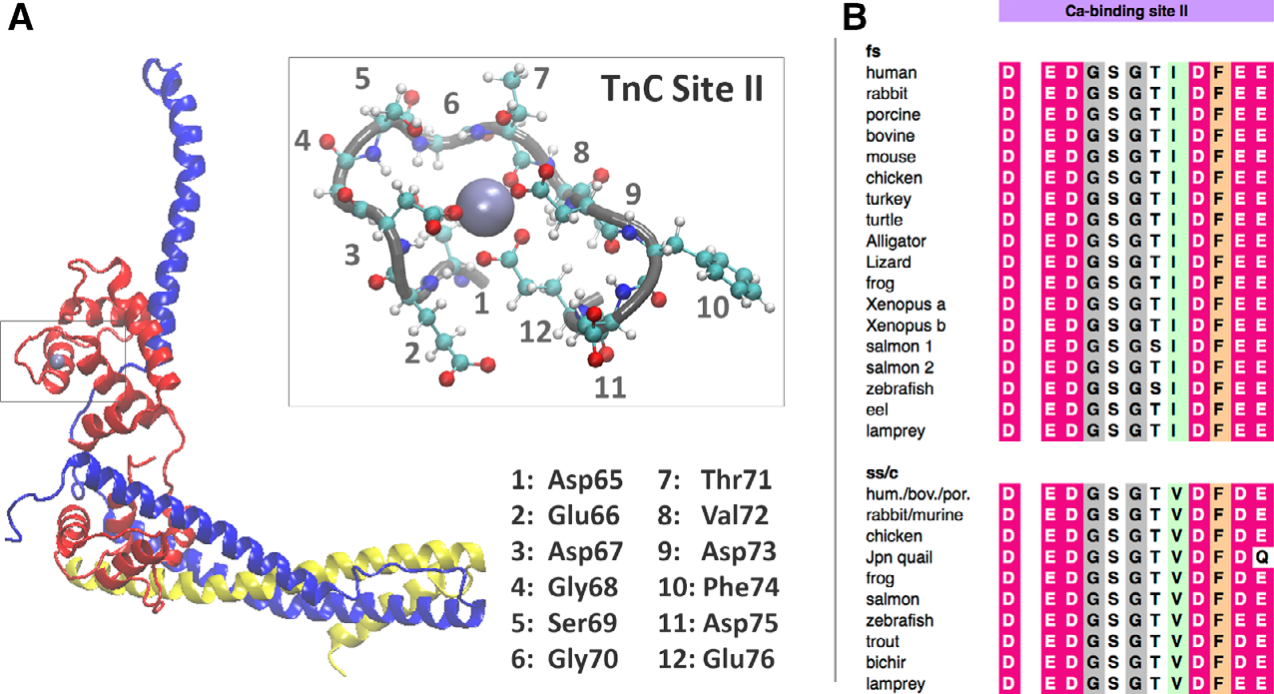
Cardiac troponin molecule TnC subunit Ca^2+^ binding site II residues and sequence conservation. (A) The thin filament Ca^2+^ sensor cardiac troponin molecule depicted as NewCartoon representation. TnI, TnC and TnT subunits are shown in blue, red and yellow. The Ca^2+^ binding site II is surrounded by a gray rectangle. In the expanded view, the Ca^2+^ binding site II is shown as a tube representation, and Ca^2+^ binding site residues are shown in the CPK representation; each residue number is shown per its position within the site, and the Ca^2+^ ion is shown in an ice‐blue vdW representation. (B) The amino acid sequence in the Ca^2+^ binding site II, including noncoordinating residues, exhibits high conservation in both cardiac and skeletal isoforms. The sequence is identical among several vertebrate TnC molecules. One notable exception is quail cardiac TnC, in which Gln76 replaces Glu. bov., bovine; c, cardiac; hum., human; por., porcine; ss, slow skeletal.

Ca^2+^ binding to the regulatory Ca^2+^ binding site(s) in TnC triggers muscle contraction through structural rearrangement of the thin filament proteins [4]. Whereas fast skeletal (fs) TnC has two functional Ca^2+^‐binding sites in the TnC N‐lobe (sites I and II), the cardiac counterpart has only one functional regulatory site, site II. This makes the investigation of Ca^2+^ binding and release events of the cardiac troponin complex more focused, because the cooperativity between the Ca^2+^ binding sites in the TnC N‐lobe need not be considered. It is believed that the N‐lobe of skeletal TnC opens its hydrophobic pocket when it binds Ca^2+^ to provide a Ca^2+^‐dependent protein interaction site [5, 6]; in contrast, the cardiac TnC N‐lobe hydrophobic pocket has been observed in closed conformation [7]. The switch region of TnI, which interacts with the hydrophobic pocket, is considered to help open it [8]. It is generally believed that the interaction of the switch region of TnI with the TnC N‐lobe hydrophobic pocket induces detachment of the inhibitory region and the second actin‐binding region from actin, and allows tropomyosin to move azimuthally on the actin filament, which in turn exposes myosin binding site(s) on actin molecules [9, 10].

Next to experimental work, recent computational investigations and methods [11] have been focused on diverse research topics, such as the structure and dynamics of troponin, Ca^2+^ regulation and structure‐based troponin‐binding small‐molecule discovery [12, 13, 14, 15]. There are three key motivations driving the investigations of the Ca^2+^‐dependent behavior and interdomain interactions of the cardiac troponin complex in detail using molecular dynamics (MD) simulations. First, a number of disease‐linked mutations have been found in the cardiac troponin components [16, 17]. Some of these mutations induce altered Ca^2+^ sensitivity of myofilament activity, which often stems from altered Ca^2+^ affinity of the TnC regulatory site in the presence of actin and tropomyosin. Interestingly, the locations of these mutations coincide with the segment where peptide backbones are more dynamic, as shown by hydrogen–deuterium exchange experiments [18]. This observation emphasizes the importance of protein dynamics to its function, although the observed timescale in hydrogen–deuterium exchange experiments is quite different from those accessible with MD simulations. Second, the knowledge of the structural details can guide the development of Ca^2+^ sensitizers and other troponin‐binding compounds [19, 20, 21] for heart disease therapy. TnC, as well as TnC–TnI interfaces, are generally targeted by cardiotonic drugs [22, 23], and even the putative agent of the ‘French paradox’, *trans*‐resveratrol, is also a TnC N‐lobe targeting compound [24]. Third, although aspects of the protein dynamics of Ca^2+^‐bound and unbound states of the N‐lobe of TnC and of the cardiac troponin complex, as well as the Ca^2+^ binding in a related protein (calmodulin), have been investigated by several groups [25, 26, 27, 28], Ca^2+^ binding events to the regulatory site of cardiac troponin have not been fully explored yet.

Previously, our work reported the detailed sequence of structural events of skeletal troponin after removing the Ca^2+^ ions from its regulatory sites [29]. In this study, we explore key aspects of the equilibrium dynamics of cardiac troponin in the apo‐state and holo‐state, investigate the path of Ca^2+^ binding into site II and describe the atomic level details of the integrated residue motions that achieve the capture and binding of the Ca^2+^ ion into the binding site. Furthermore, the removal of the TnI switch region from the TnC N‐lobe hydrophobic pocket is simulated, and subsequently the dynamics of the hydrophobic pocket and a set of key TnI‐TnC interdomain interactions are described.

## Materials and methods

### Molecular model preparation

The crystal structure coordinate file of human cardiac troponin Ca^2+^‐saturated form molecule (PDB: 1J1E) [30] was obtained from the Research Collaboratory for Structural Bioinformatics (RCSB) Protein Data Bank (PDB) [31]. Using this structure as a starting point, we prepared a structural model of the Ca^2+^‐saturated cardiac troponin core domains, correcting for the missing residues that are not present in the PDB: 1J1E file. From the PDB: 1J1E coordinate file, chains D, E and F and the Ca^2+^ ions (chains D, E and F of PDB: 1J1E correspond to TnC, TnT and TnI) were obtained. The gaps in these chains were filled using known structures of troponin as follows: residues 49 and 50 from chain A of PDB: 1JIE (which is also TnC) were extracted and inserted into chain D to complete the gap in chain D. Using skeletal troponin crystal structure coordinate file PDB: 1YTZ [32] as a source, we extracted residues 104–114 of TnI and inserted them into chain F to complete the gap in residues 137–147 in cardiac TnI, which are missing in PDB: 1J1E chain F. Residue 143 in the model cardiac TnI structure was changed to threonine from the proline that is found in PDB: 1YTZ at this position. Coordinates of cardiac PDB: 1JIE chain C residues 35–40 were used to fill in another missing segment in the F chain. The final production model system included also the C‐terminal extension of TnI residues 150–190.

Protein structure files were created using the molecular modeling package VMD [33] and the plug‐in program *psfgen*. Hydrogen atoms were added, and the protein system was solvated in an explicit solvent environment. The *CHARMM* [34] force field was used for the protein; water was considered as the TIP3P model [35]. The total system size for the molecular system was ~ 190 000 atoms. The molecular system was energy minimized by conjugate gradient method and gradually heated to 300 K.

### Equilibrium and steered MD simulations

MD simulations of the prepared core cardiac troponin molecule model were computed using the namd [36] software package. Periodic boundary conditions in the NPT ensemble were applied; temperature and pressure were kept constant by using Langevin dynamics at 1 atm and 300 K. Electrostatic interactions were computed by Particle mesh Ewald method. Nonbonded interactions were treated with a cutoff using a switching function beginning at 10 Å and reaching zero at 14 Å. Overall, 20 simulations for a total duration of 0.45 μs of simulation time were performed.

Equilibrium MD simulations were performed of the system in the Ca^2+^‐bound state (before Ca^2+^ release), in the Ca^2+^‐depleted state (after force extraction of the Ca^2+^ ion from site II), during Ca^2+^ binding, and in the Ca^2+^‐bound state (after Ca^2+^ binding). Steered molecular dynamics (SMD) simulations [37, 38], which is a computational method wherein a predetermined force is applied in specific magnitude and direction to a predetermined atom (force clamp mode) or a specific atom is translated in a predetermined velocity (constant velocity mode), were performed with the goal to extract the Ca^2+^ ion form site II. During these SMD simulations, the TnC N‐lobe was harmonically constrained, and the Ca^2+^ ion was pulled away at 10 m·s^−1^ until it was translated ~ 40 Å from the original location. The final coordinates of the SMD simulations were used as a starting point for equilibrium MD simulations of the behavior of cardiac troponin in the Ca^2+^‐depleted state and the binding of the Ca^2+^ ion into the binding site.

Using SMD and an equilibrated structure of Ca^2+^‐depleted cardiac troponin, the TnI switch region was extracted from the TnC N‐lobe hydrophobic pocket. The point‐of‐pulling atom was selected as the alpha‐C atom of TnI residue 146; harmonic restraints were applied to the alpha‐C atoms of the TnC N‐lobe hydrophobic pocket or to all atoms of the hydrophobic pocket. The pulling atom was translated at 1 m·s^−1^ in the approximate direction of TnT residue 224. Coordinates extracted from two distinct TnI switch extraction stages from the SMD simulations were used as the starting point for equilibrium MD simulations of the behavior of TnI, TnC and TnI–TnC interdomain interactions.

### Analysis and visualization

MD simulation data extraction, trajectory analysis, figures and visualizations were completed using VMD and MATLAB. Sequences of TnC were compared previously [39]. The comparison was extended to all available sequences of TnC, and the conservativeness of each position along the sequence was analyzed. Sequence comparison of different sequences was carried out using blast and CLUSTLAW, followed by a visual examination. The degree of conservation was measured by sequence entropy [40].

## Results and Discussion

First, key dynamic features and interactions in the Ca^2+^‐saturated state of cardiac troponin are characterized, and then the atomic level details of the coordinated residue motions that result in the capture, translation and binding of the Ca^2+^ ion into the binding site II of TnC are described. Second, the TnI switch region displacement from the TnC N‐lobe hydrophobic pocket and a set of key TnI–TnC interdomain interactions are investigated.

### Key features of the equilibrium dynamics of the Ca^2+^‐saturated state of cardiac troponin TnC N‐lobe

The TnC Ca^2+^ binding site II consists of 12 residues (Fig. 1A): Asp65 (aspartic acid), Glu66 (glutamic acid), Asp67 (aspartic acid), Gly68 (glycine), Ser69 (serine), Glu70 (glutamic acid), Thr71 (threonine), Val72 (valine), Asp73 (aspartic acid), Phe74 (phenylalanine), Asp75 (aspartic acid) and Glu76 (glutamic acid). The sequence of the binding site, including noncoordinating residues, is identical among all the vertebrate TnC (both cardiac and skeletal isoforms), with one exception in quail cardiac TnC, in which Gln76 replaces Glu (Fig. 1B). In the holo‐state, the Ca^2+^ ion is grasped by its coordinating ligands in the binding site: it is coordinated by the residues at positions 1, 3, 5 (via water), 7, 9 and 12 [41, 42]. The 12th position of the site is a bidentate ligand in the form of a conserved Glu residue. During the holo‐state MD simulations, the Ca^2+^ ion was well settled into the coordination space formed by the ligands, and the coordination distance between the Ca^2+^ ion and its ligands was well maintained. In the MD simulations, the distance of the Ca^2+^ ion to the main‐chain oxygen of residue Thr71O was maintained at ~ 2.5 Å, to the bidentate ligand Glu76 at ~ 2.7 Å and to the monodentate ligands Asp65, Glu67 and Asp73 at ~ 3.3 Å (Fig. S1).

A set of interactions occurs in the TnC site II, realized between the Ca^2+^ ion, side‐chain OE^1^ and OE^2^ of Glu12 (Glu76) and the backbone N atoms of the residues at positions 2 and 9 (Glu66 and Asp73) (denoted here as N^2^‐OE^12^/N^9^‐OE^12^), which are particularly important for the regulatory function of the site, as analogously observed in fs troponin [29]. The importance of this interaction for the performance of the regulatory function of the E‐F hand motif in calmodulin was shown in experimental studies where the Glu was mutated to Gln [43, 44]. Other Nuclear Magnetic Resonance (NMR) work [45] and Circular dichroism (CD) analyses [46] have unveiled the fact that mutation of the Glu to Ala induces the N‐lobe of the TnC subunit to remain in closed‐like state and impairs the functionality of the protein even if Ca^2+^ is present in high concentration. In the Ca^2+^‐saturated state, the side‐chain oxygen atoms of the Glu12 are positioned toward the inside of the Ca^2+^ binding site, holding the Ca^2+^ ion in a bidentate charge–charge interaction while at the same time participating in H‐bonding with the main‐chain nitrogen atoms of the two residues at positions 2 and 9. In the Ca^2+^‐saturated state, the N^2^‐OE^12^/N^9^‐OE^12^ interaction distances were well maintained throughout the simulations (Fig. S2). These measurements give an indication that the binding site is closed around the Ca^2+^ ion.

During the cardiac troponin structure modeling process, the C‐terminal extension of TnI, which is resolved in PDB: 1J1E, was incorporated in our model. This segment of TnI, which has bent conformation in the experimentally derived coordinates, transitioned rapidly to a straight helix conformation during the MD simulations (Fig. S3).

### Extraction of Ca^2+^ from site II and site II relaxation

**Fig. 2:**
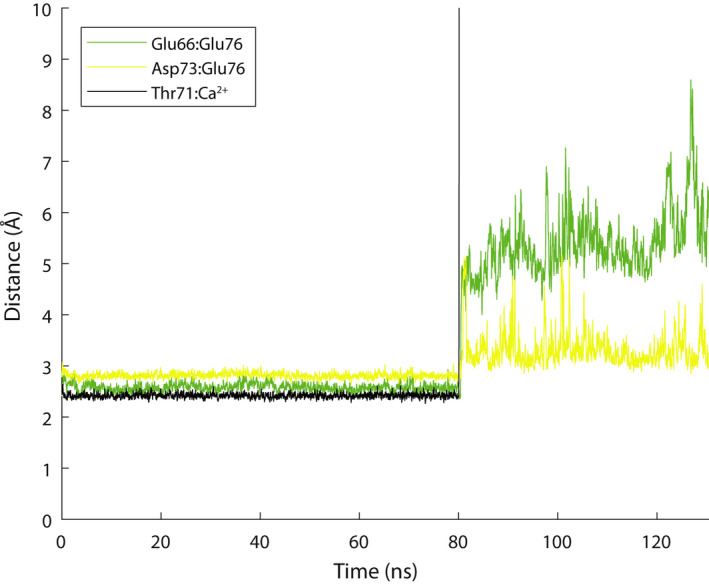
Ca^2+^‐saturated and ‐depleted state behavior of TnC site II N^2^‐OE^12^/N^9^‐OE^12^ interaction. Dynamic behavior of TnC site II N^2^‐OE^12^/N^9^‐OE^12^ interaction during 80 ns of Ca^2+^‐saturated state equilibrium MD simulation, followed by extraction of the Ca^2+^ ion from site II by SMD, followed by >40 ns of Ca^2+^‐depleted state equilibrium MD simulation. The distances between 12/Glu76OE:2/Glu66HN shown in green and 12/Glu76OE:9/Asp73HN shown in yellow. Distance of Ca^2+^ to atom Thr71O is shown in black to highlight the Ca^2+^ state of site II. Using SMD, after *t* = 80 ns, the Ca^2+^ is extracted and pulled away to 40‐Å distance from site II. The N^2^‐OE^12^/ N^9^‐OE^12^ interaction is well maintained in the Ca^2+^ bound state but is immediately released and does not re‐form in the Ca^2+^‐depleted state.

Using SMD pulling, we extracted the Ca^2+^ ion from the regulatory site II. The protein was kept in place by harmonic constraints, and the Ca^2+^ ion was pulled at constant velocity of 10 m·s^−1^. After Ca^2+^ was displaced to a distance of ~ 40 Å, the steering force was no longer applied. Ca^2+^ removal from the binding site was followed by the immediate breakage of the N^2^‐OE^12^/N^9^‐OE^12^ H‐bond interactions. This enhances the motional degrees of freedom at the N‐terminal end segment of the Ca^2+^‐binding loop and uncouples it from the C‐terminal end segment, which allows positions 1 and 12 to separate from each other. Within site II, the Glu76 ligand side chain moves away from the inside of the site. The combined conformational change after release of Ca^2+^ is a subtle expansion of the binding site as observed in fs TnC [29]. The N^2^‐OE^12^/N^9^‐OE^12^ interaction does not re‐form without the Ca^2+^ ion in the site (Fig. 2). As the N^2^‐OE^12^/N^9^‐OE^12^ H‐bonds are released and the binding site expands, the side chains of ligands 1, 3, 9 and 12, which have previously been held in toward the ion, are now free to move away and repoint into the solvent. During the Ca^2+^‐depleted state simulations, release of the TnI switch region from the N‐lobe of TnC was not observed, because these events are slow processes and may take place in millisecond order [47]. Binding of the inhibitory region, which precedes the switch region of TnI, to actin facilitates a release of the switch region from TnC ~ 10 times faster.

Having extracted Ca^2+^ from site II of this system, there are now complete snapshots of four possible structural configurations of the complex of TnC–Ca^2+^, and the switch region of TnI during Ca^2+^ activation–relaxation. These states are: (a) apo‐TnC without TnI (PDB: 1SPY), (b) Ca^2+^–TnC without TnI region bound (PDB: 1AP4), (c) apo‐TnC with TnI (this work), and (d) Ca^2+^–TnC with TnI bound (PDB: 1J1D, PDB: 1J1E and this work). In (a) and (b), however, only the N‐lobe of TnC and the peptide that corresponds to the switch region of TnI were used for structural determinations instead of the whole cardiac troponin core domain. Because the location and structure of the unbound switch region has yet to be revealed, the detailed intramolecular and intermolecular interactions found in these structures may not be the same as those in the core domain structure.

### Mechanism of Ca^2+^ binding into TnC site II

**Fig. 3:**
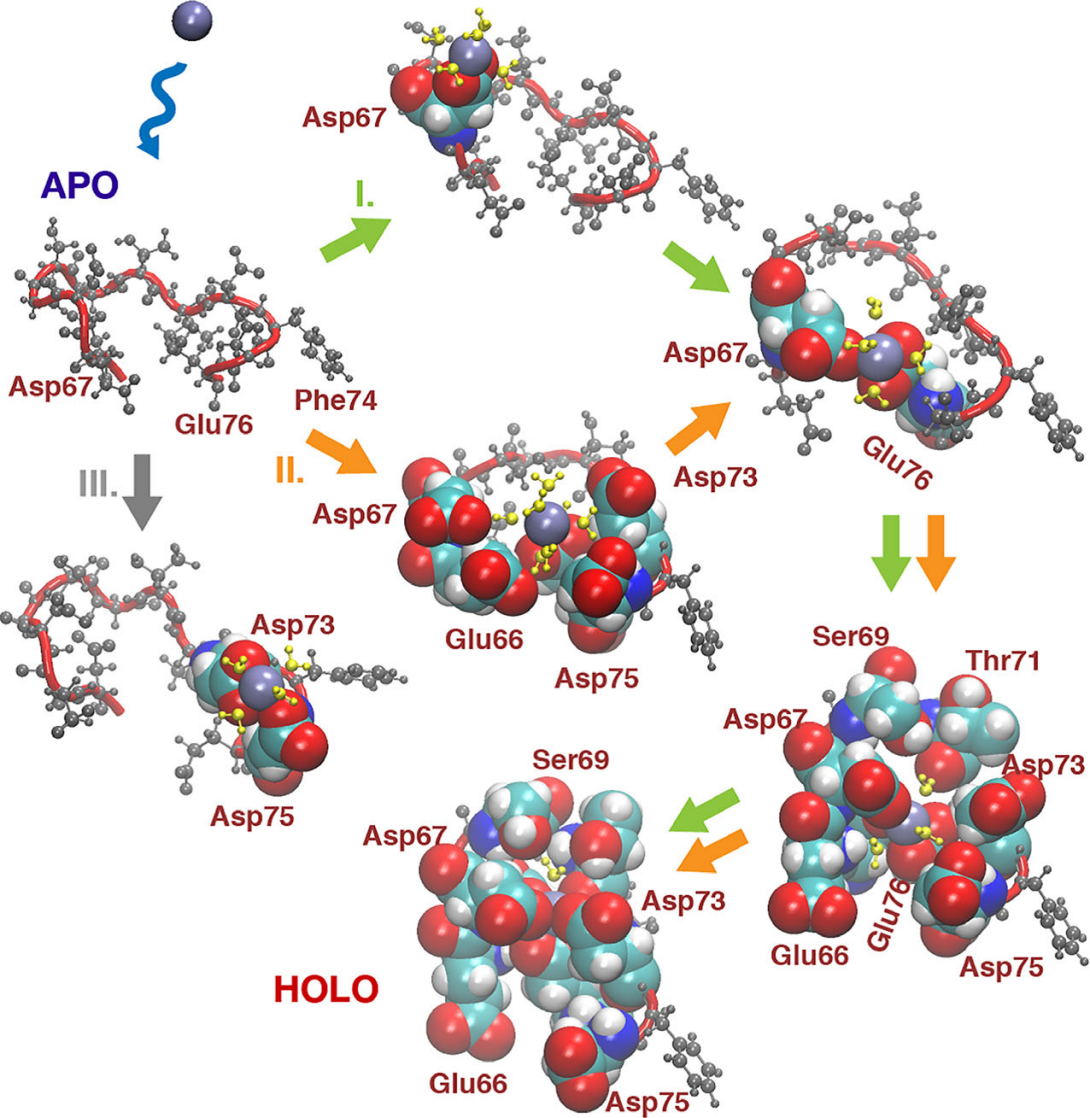
Binding of Ca^2+^ into the TnC N‐lobe regulatory site II. (a) Capture: Glu66, Asp67, Asp73, Asp75 capture the floating Ca^2+^ in a cooperative motion (orange arrow), but the Ca^2+^ ion also may be captured directly by ASP67 in bidentate mode (green arrow). If Asp75 captures the Ca^2+^ ion, it may get trapped between residues Asp75 and Asp73 and was not transposed into the binding site (gray arrow) during the duration of the simulation. (b) Toward Ca^2+^ coordination: Asp67 (evolving from bidentate to monodentate coordination) cooperatively with Glu76 (evolving from monodentate to bidentate coordination) transpose the ion inward; the binding site starts closing around the Ca^2+^, while Phe74 serves as an anchor; Asp66 and Glu75 serve as gatekeepers so the Ca^2+^ does not escape back into the solvent. Asp65 and Thr71O also are now close to Ca^2+^. A water molecule present inside the site hinders those residues from joining the coordination set. (c) Expulsion of the water molecule: Ca^2+^ is positioned away from the outside solvent environment when the water molecule is expelled from the site into the outside cavity by switching places with the Ca^2+^ ion; Asp65 and Thr71O also start coordinating the Ca^2+^ ion, coincident with the final decrease in the Ca^2+^‐to‐71O distance. (d) Ca^2+^ is bound in site II: coordination of Ca^2+^ is Asp65, monodentate by side‐chain oxygen; Asp67 monodentate, by side‐chain oxygen; Ser69, at longer distance, monodentate by side‐chain oxygen, residue Thr71 twice (monodentate by side‐chain oxygen and main‐chain oxygen); Asp73 monodentate by side‐chain oxygen; and Glu76 bidentate by side‐chain oxygen atoms. The side‐chain coordination of residues 69 and 71 is via a water molecule that also plugs into the outside of the coordination cavity.

**Fig. 4:**
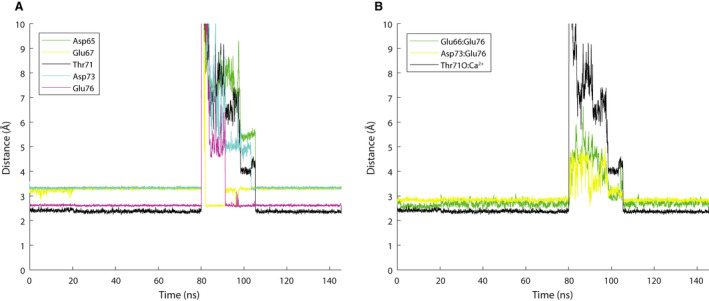
Key distances within the Ca^2+^ binding site in holo‐state and apo‐state. (A) Coordination distance between the Ca^2+^ ion and site II residues. Evolution from holo‐state to apo‐state and back to holo‐state. Distances of the Ca^2+^ ion to the coordinating ligands are depicted. Starting from *t* = 80 ns, the Ca^2+^ ion is extracted via SMD from the binding site but binds back after force application is stopped. Initially, it is captured by Glu67 (yellow line) as evidenced by the reduction in separating distance, and Glu67 starts in bidentate coordination followed by cooperation with Glu76 (purple line). Glu67 switches to monodentate (jump in yellow line, drop in purple line); also, at ~ 97 ns, the two ligands briefly switch coordination mode. The final big decline in distance to Thr71O (to ~ 2.5 A) and Asp65 is when the Ca^2+^ ion and the water molecule switch places and the coordination plane around the ion is finally closed. (B) N^2^‐OE^12^/N^9^‐OE^12^ H‐bond interaction distances. Evolution from holo‐state to apo‐state and back to holo‐state. In the Ca^2+^‐saturated state (until *t* = 80 ns), the N^2^‐OE^12^/N^9^‐OE^12^ distances are well maintained. Removal of Ca^2+^ causes the N^2^‐OE^12^/N^9^‐OE^12^ interactions to immediately disband. Binding of the Ca^2+^ ion into the site causes the interactions to re‐form (post ~ 110 ns). Distance between 12/Glu76OE:2/Glu66HN shown in green and 12/Glu76OE:Asp73HN shown in yellow. Distance of the Ca^2+^ to residue Thr71O (black) is shown to highlight the Ca^2+^‐bound state.

The binding of Ca^2+^ into the site II binding loop was observed to occur via different modes (Fig. 3, flows I, II and III), which differed by how the Ca^2+^ was captured and if the ion was fully re‐bound in the regulatory site. In this section, the sequence of coordination events and the mechanism of Ca^2+^ ion binding in the regulatory site in cardiac troponin are described. Figure 3 depicts the sequence of events observed in the binding of Ca^2+^ into the binding site, whereas Fig. 4 depicts the dynamic behavior of separating distances from key site II residues to the Ca^2+^ ion (Fig. 4A) and the N^2^‐OE^12^/N^9^‐OE^12^ H‐bond interaction distances (Fig. 4B).

#### Capture of the Ca^2+^ ion

The first step of Ca^2+^ binding into site II is the Ca^2+^ ion capture. Several residues (Glu66, Asp67, Asp73 and Asp75) are well positioned to capture the Ca^2+^ ion. In our simulations, these residues were observed catching the passing ion. Initial capture was observed by position 3 (Asp67) residue onto Ca^2+^ in a bidentate coordination (Fig. 3, flow I; Video S1). Initial capture was also observed by residues Glu66, Asp67, Asp73 and Asp75 in a remarkable movement of cooperation, and then the ion was passed onto Asp67 and Glu76, which led it deep into the site (Fig. 3, flow II). In addition, initial capture of the ion was also observed by residue Asp75, followed by a cooperative move toward residue Asp73 (Fig. 3, flow III). In this case, however, the Ca^2+^ ion remained trapped between residues Asp73 and Asp75 and did not bind into the site for the duration of the simulation (80 ns). Thus, it appears that the approach vector of the Ca^2+^ ion is also important. If the Ca^2+^ ion is caught by the N‐terminal segment of the binding site or the four front residues in concert, it quickly is transposed and bound in the site. If, however, the ion is caught by the C‐terminal segment of the site, the ion may become trapped between residues Asp73 and Asp75, in which case binding is delayed or perhaps does not occur.

#### Toward Ca^2+^ coordination

After a Ca^2+^ ion is caught by site II residues, the process realizing full coordination continues by site residues maneuvering of the Ca^2+^ ion deeper in the binding site and the tightening of the loop around the Ca^2+^ ion. In this step, the key factor is the cooperation between residues Asp67 and Glu76. These residues work in unison to bring the ion farther in the site. In this process, the coordination of Asp67 evolves from bidentate to monodentate, whereas the coordination of Glu76 evolves from monodentate to bidentate (Fig. 3). At the same time, residues Glu66 and Asp75 act to prevent the Ca^2+^ ion from jumping out of the site. During this step, residues Asp65 and Thr71 are not at their nearest possible distance to the Ca^2+^ ion, and a water molecule is also present in the site and occupies the space between the Ca^2+^ ion and Thr71 main‐chain O_2_ atom.

#### Final step: water molecule expulsion

In the final step of the binding process, the water molecule is expelled from the binding site, and the site is closed around the Ca^2+^ ion; the water molecule and the Ca^2+^ ion essentially switch places. Ca^2+^ is thus buried away from the solvent when the water molecule is expelled from the site into the outside cavity (Fig. 3). This final step of the ion binding is marked by the decrease of overall coordination distance (more specifically the decrease in the distance between Thr71O and the Ca^2+^ ion) allowed by the expulsion of the water molecule. Residues Asp65 and Thr71 also make close contact with the ion, which is coincident to the final decrease in the distance from Ca^2+^ ion to Thr71O and Asp65 (Fig. 4A). While the ion is being moved deeper into the binding site, the H‐bond interaction N^2^‐OE^12^/N^9^‐OE^12^ re‐forms (Fig. 4B). As evidenced by the plot, while the ion is positioned in the site, the N^2^‐OE^12^/N^9^‐OE^12^ interaction re‐forming causes a tightening of the Ca^2+^ binding loop. The site expansion previously discussed is reversed, and the site is now contracted around the Ca^2+^ ion.

The conformation and coordination of the ion observed after binding is consistent with our observation in the holo‐state MD. It is realized by the following conformational participants: residue Asp65, monodentate via side‐chain oxygen; residue Asp67, monodentate via side‐chain oxygen; residue Ser69, monodentate via water and side‐chain oxygen; residue Thr71 twice (first by side‐chain oxygen via water molecule and 71 main‐chain oxygen); residue Asp73 monodentate via delta oxygen and residue Glu76 bidentate via delta oxygen. The side chain coordination of residues Ser69 and Thr71 is via a water molecule that plugs into the coordination cavity (Fig. S4). In this conformation, the Ca^2+^ is buried away from the solvent and surrounded by six oxygen atoms, another oxygen atom from the water molecule is also in contact with the ion, and two more oxygen atoms (residues Ser69 and Thr71) are in H‐bond interaction with the water oxygen atom. The fact that final coordination conformation of Ca^2+^ and the final decline of the distance to Thr71O are realized at the expulsion of the water molecule suggests that the escape route for Ca^2+^ on decline in Ca^2+^ concentration may be through the cavity that is plugged in by the water molecule (Fig. S5).

The driving force for this three‐step binding process of Ca^2+^ ion is effected by electrostatic steering; the Ca^2+^ ion, once captured, is attracted by the other large negative charges that are in proximity within the binding site. Thus, changes in the binding site or the surrounding electrostatic niche may have profound physiological implications. For example, it is well known that the thin filaments with hypertrophic cardiomyopathy‐linked mutations occurring in the C‐terminal mobile domain of TnI, which is believed to be located close to the N‐terminal regulatory lobe of TnC [48, 49], show higher affinity for Ca^2+^ [50]. The mobile domain of TnI is abundant in basic residues. Mutations of these basic residues to nonbasic amino acids may quite be the causative factor of the higher affinities of the thin filaments with hypertrophic cardiomyopathy‐linked mutations for Ca^2+^ by increasing the on‐rate of Ca^2+^ binding.

### SMD simulations of cardiac troponin TnI switch region displacement from the TnC N‐lobe hydrophobic pocket

It has long been considered that displacement of the TnI switch region from the TnC N‐lobe hydrophobic pocket is a required step in the calcium‐dependent regulation of troponin and the subsequent downstream transduction of the regulatory signal. To investigate the feasibility of such a mechanism, we explored the displacement of the TnI switch region from the TnC N‐lobe hydrophobic pocket by using SMD. The switch region was able to be extracted in simulation models where harmonic restraints were used within the hydrophobic pocket (either on all atoms or only on the alpha‐C atoms). Simulations without harmonic restraints resulted in the deformation of the TnC–TnI complex but no extraction of the switch region. In one SMD simulation, harmonic restraints were applied to the C‐alpha atoms of the TnC N‐lobe hydrophobic pocket, and the switch was extracted from it until the point‐of‐pulling atom translated ~ 20 Å (Fig. S6A). In the second extracting simulation, harmonic restraints were applied to all atoms of the TnC N‐lobe hydrophobic pocket, and the switch was extracted from it until the pulling atom translated ~ 60 Å (Fig. S6B).

### TnI–TnC interdomain interactions modulate the dynamics of the TnC N‐lobe hydrophobic pocket

**Fig. 5:**
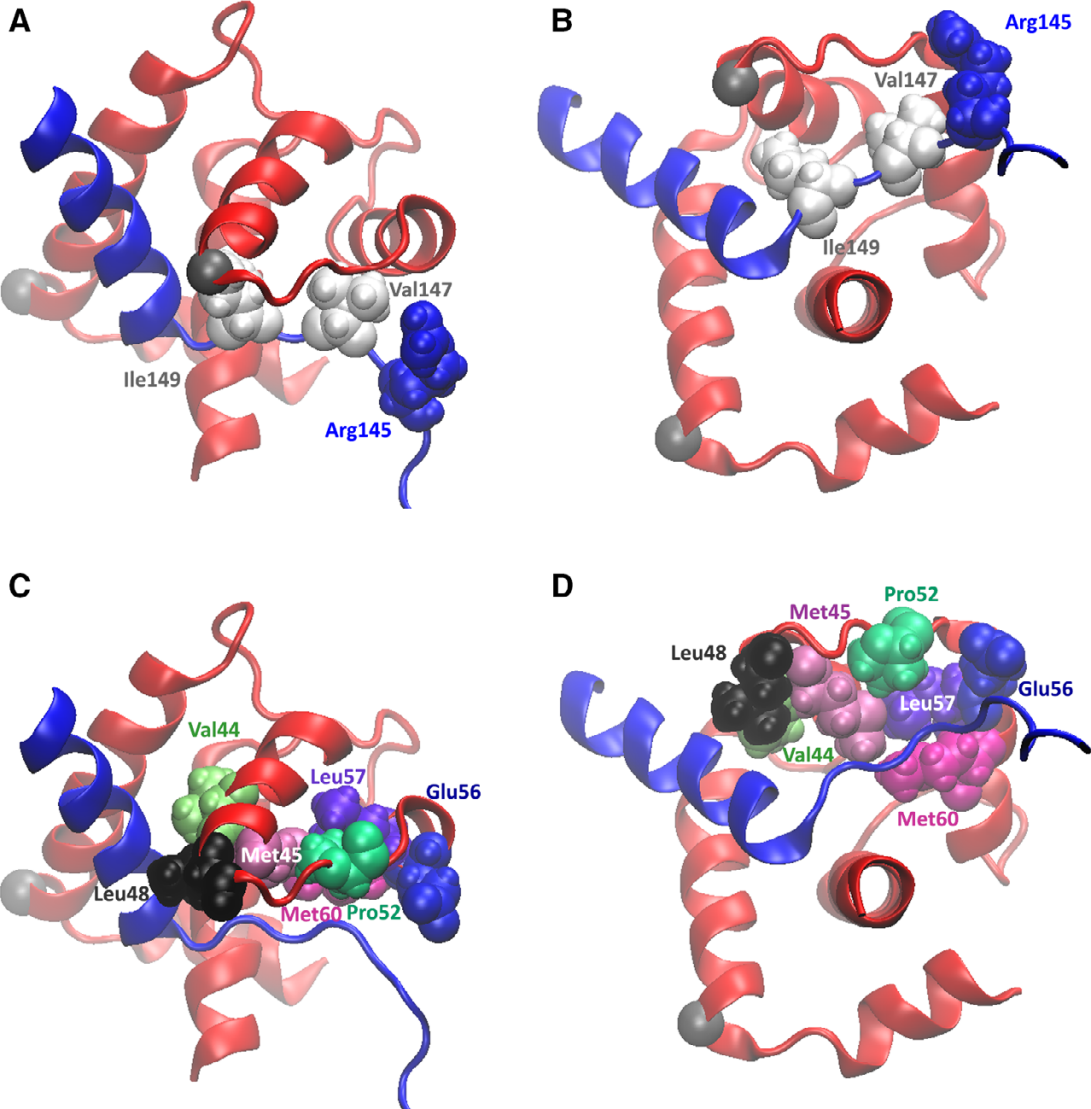
Key residues involved in TnC–TnI interdomain interaction. (A, B) Focus on TnI: hydrophobic amino acids Val147 and Ile149 and the positively charged amino acid Arg145 participating in interdomain interaction with TnC residues. The TnI chain is shown in blue and NewCartoon representation, and the TnC chain is shown in red and NewCartoon representation. Alpha‐C atoms of TnC residues Glu15 and Leu48 of the TnC N‐lobe hydrophobic pocket opening are shown as gray vdW spheres. (B) Ninety‐degree rotation of (A). (C, D) Focus on TnC: hydrophobic residues Val44, Met45, Leu48, Pro52, Glu56, Leu57 and Met60 participating in interdomain interaction with TnI residues. The TnI chain is shown in blue and NewCartoon representation, and the TnC chain is shown in red and NewCartoon representation. Alpha‐C atoms of TnC residues Glu15 and Leu48 of the TnC N‐lobe hydrophobic pocket opening are shown as gray vdW spheres. (C) Ninety‐degree rotation of (D).

**Fig. 6:**
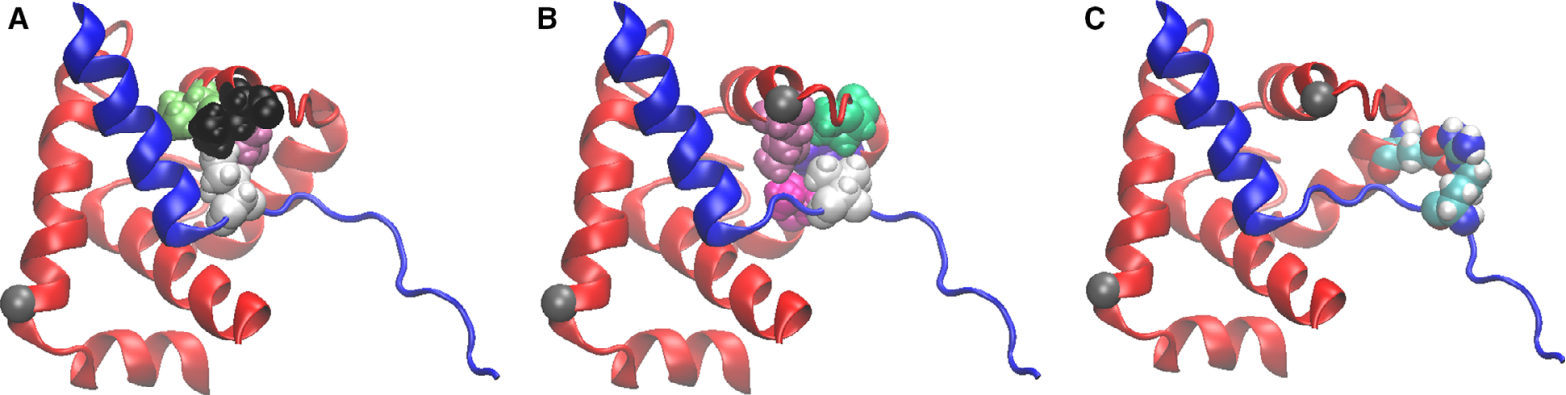
Interdomain interaction between TnC and TnI. (A) Hydrophobic interaction *i1* between TnI residue Ile149 (shown in white) and TnC residue Val44 (shown in green), TnC residue Met45 (shown in purple) and TnC residue Leu48 (shown in black) (TnI residue Ile149 is surrounded by nonpolar residues). (B) Hydrophobic interaction *i2* between TnI residue Val147 (shown in white) and TnC residue Met45 (shown in purple), TnC residue Pro52 (shown in green), TnC residue Leu57 (shown in blue) and TnC residue Met60 (shown in pink). TnI residue Val147 is surrounded by nonpolar residues. (C) H‐bond interaction *i3* between TnI residue Arg145 (shown in atom colors) and TnC residue Glu56 (shown in atom colors).

Examination of the TnI and TnC dynamic behavior during the simulation trajectories reveals a network of interdomain interactions that have the potential to modulate the opening and closing of the TnC N‐lobe hydrophobic pocket and the ability of the TnI switch region to translate out and back into it, thus impacting the Ca^2+^‐driven regulation of cardiac muscle contractility. Three key interactions occur between residues of TnC and TnI. More specifically, TnI residues involved are two hydrophobic residues, Val147 and Ile149; and one positively charged residue, Arg145 (Fig. 5A,B); and TnC residues‐Val44, Met45, Leu48, Pro52, Glu56, Leu57 and Met60 (Fig. 5C,D). The interdomain interactions observed are as follows: (*i1*) hydrophobic TnI residue Ile149 is positioned into a hydrophobic niche formed by TnC residues Val44, Met45 and Leu48 (Fig. 6A); (*i2*) hydrophobic TnI residue Val147 is positioned into a hydrophobic niche formed by TnC residues Met45, Pro52, Leu57 and Met60 (Fig. 6B); and (*i3*) an H‐bond interaction takes place between TnI residue Arg145 and TnC residue Glu56 (Fig. 6C).

**Fig. 7:**
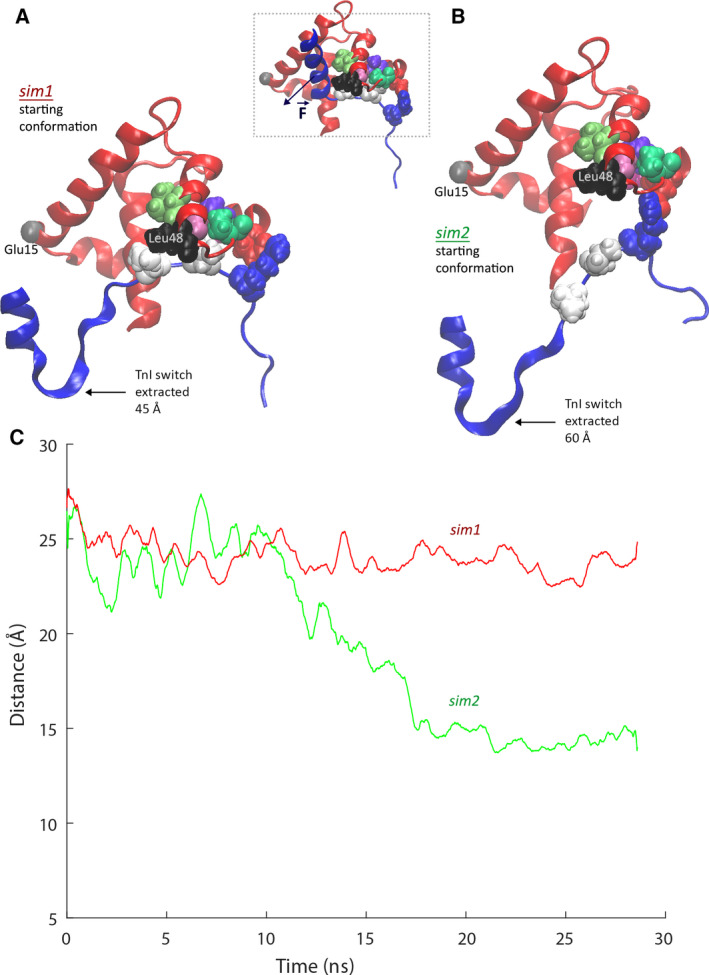
Starting conformation for TnI switch region SMD extraction, the two distinct switch extraction conformations used for post‐extraction equilibrium MD *sim1* and *sim2* simulations, and evolution of the TnC N‐lobe hydrophobic pocket opening distance in *sim1* and *sim2*. Inset: starting conformation TnI switch extraction by SMD. The TnI switch is embedded in the TnC N‐lobe hydrophobic pocket. Residues involved in TnI–TnC interdomain interaction are shown as vdW spheres; force application is donated by F. (A) Starting conformation for post‐TnI switch extraction equilibrium MD simulation *sim1* after translation of ~ 45 Å of the pulling atom from the starting conformation is achieved by SMD. The TnI switch is pulled from the TnC N‐lobe hydrophobic pocket; however, the three TnC–TnI interactions (*i1, i2* and *i3*) are maintained. (B) Starting conformation for post‐TnI switch extraction equilibrium MD simulation *sim2* after translation of ~ 60 Å of the pulling atom from the starting conformation is achieved by SMD. The TnI switch is pulled from the TnC N‐lobe hydrophobic pocket, TnI residues Ile149 and Val147 are extracted from the hydrophobic niches in TnC (interactions *i1* and *i2*), while the H‐bond interaction (*i3*) by TnI residue Arg145 and TnC residue Glu56 (*i3*) is still maintained. (C) Distance between alpha‐C of residues Glu15 and Leu48 of the TnC N‐lobe hydrophobic pocket in *sim1* and *sim2*. The TnC N‐lobe hydrophobic pocket remains open (distance ~ 25 Å, red line) in *sim1*. The TnC N‐lobe hydrophobic pocket closes rapidly in *sim2* (green line).

To explore the dynamic behavior and effect of interactions *i1*, *i2* and *i3*, we performed equilibrium MD simulations from two distinct conformational starting points that represented distinct SMD displacements of the TnI switch from the TnC N‐lobe hydrophobic pocket. The first simulation (*sim1*) started from a conformation of 45 Å translation of the TnI SMD pulling atom with the TnI switch region displaced, but interdomain interactions *i1*, *i2* and *i3* maintained (Fig. 7A). The second simulation (*sim2*) started from a conformation of 60 Å translation of the TnI SMD pulling atom and the switch region displaced from the TnC N‐lobe hydrophobic pocket but with the hydrophobic interdomain interactions (*i1*, *i2*) disrupted, whereas the H‐bond interaction between TnC residue Arg145 and TnI residue Glu56 (*i3*) was allowed to remain strained (Fig. 7B).

In *sim1*, the TnC N‐lobe hydrophobic pocket remained in an open state at ~ 25 Å distance (measured between alpha‐C of residues Glu15 and Leu48, located in the TnC N‐lobe hydrophobic pocket) (Fig. 7C, red line); in the *sim2* simulation, the pocket closed swiftly to a distance of ~ 15 Å (measured between alpha‐C of residues Glu15 and Leu48, located in the TnC N‐lobe hydrophobic pocket) (Fig. 7C, green line). In *sim2*, the H‐bond interaction *i3* between TnI residues Arg145 and TnC Glu56 continuously separated and re‐formed. Absence of the hydrophobic interactions *i1* and *i2* allowed the closure of the TnC N‐lobe hydrophobic pocket, whereas if the interactions were present, the pocket remained open. Therefore, it is feasible to consider that modifications of these interactions may modulate the ability of the TnC N‐lobe hydrophobic pocket to close and open. The H‐bond interaction *i3* does not need to be broken for the pocket to close; thus, it can be hypothesized that this H‐bond interaction serves to stabilize the connection between TnI and TnC and may serve to anchor the TnI switch region nearby the hydrophobic pocket after removal to facilitate subsequent reentry. The removal of TnI residues Val147 and Ile149 from the hydrophobic space may also be the mechanism that drives the closure of the hydrophobic pocket, by creating an open hydrophobic space that is then followed by the translation of the TnC hydrophobic residues involved in interaction with TnI residues Val147 and Ile149.

It should be mentioned that mutations at TnI residue Arg145 are linked to hypertrophic or restrictive cardiomyopathy. Arg145 is believed to be involved in actin interaction, as well in the process of muscle regulation. Thus, it may not be possible to interpret functional consequences of mutations at Arg145 in a straightforward way [51]. Yet a decrease in myofilament activity in the presence of Ca^2+^ caused by introducing mutations at this site seems to be consistent with our observation that removal of the Arg145 side chain may destabilize the Ca^2+^‐bound form of cardiac troponin [44, 47, 48].

Also, it is noteworthy that protein kinase A (PKA)‐dependent phosphorylation affects the interaction between the switch region of TnI and the N‐lobe of TnC. The microenvironment of PKA‐dependent phosphorylation sites is also affected by the presence of TnT. Thus, in a cardiac troponin system, the interaction of the TnI switch region and the N‐lobe of TnC is modulated by a number of factors [52, 53, 54].

## Summary and Significance

In this work, the dynamics of key interactions of the TnC N‐lobe were highlighted, and the atomic level details of residue coordination and cooperativity leading to Ca^2+^ capture and binding in the binding site, which would constitute the first step in a contraction signal, were described. Ca^2+^ binding was observed as a three‐step process commencing with Ca^2+^ initial capture, followed by interplay between residues that guides the Ca^2+^ ion into the binding site. At the third and final step, the Ca^2+^ ion exchanges places with a water molecule already present in the binding site, to achieve final close contact with the six oxygen atoms that coordinate it. SMD extraction of the TnI switch from the hydrophobic pocket was also performed, and the role of interdomain interaction in its closing was explored.

Cardiac troponin, which is one of the proteins of the sarcomere, plays an important role in the Ca^2+^‐dependent regulation of cardiac contractility. Diverse and precise intermolecular interactions realize the regulatory conformational response to the fluctuation of Ca^2+^ levels in cardiomyocytes and are integral to the performing of the function of the heart muscle. To this day, hundreds of mutations in proteins in the cardiac sarcomere are known to cause types of inherited cardiomyopathy, and more than 60 mutations in cardiac troponin subunits have been found in dilated, hypertrophic and restrictive cardiomyopathy. These mutations are likely to have functional consequences and be one of the primary mechanisms of cardiomyopathy pathogenesis. For basic science to enable the development of effective therapies in a clinical setting, a detailed and precise understanding of the form and function of this system is necessary. Thus, the troponin system must be fully explored and understood to comprehend the molecular mechanisms underlying disease and to help develop therapy.

## Conflict of interest

The authors declare no conflict of interest.

## Author contributions

GZG, MK, TK and HL designed the study. GZG, MK, TK and HL performed the experiments, collected and processed the data, and implemented the analysis. GZG, MC, TK and HL wrote and revised the manuscript. All authors approved the content and the submission of the manuscript.

## Supporting information


**Video S1.** Ca^2+^ binding into TnC site II.
Figure S1: Coordination of Ca^2+^ in Site II
Figure S2: Ca^2+^ saturated state of troponin: N^2^‐OE^12^/N^9^‐OE^12^ interaction
Figure S3: Straightening of TnI C‐terminal α‐helical extension
Figure S4: The achieved coordination of Ca^2+^ after rebinding
Figure S5: Escape route of the Ca^2+^ ion
Figure S6: Extracting the TnI switch region from the TnC A/B hydrophobic pocket by using SMD

## Data Availability

Model structural coordinates are available on request from the corresponding authors.
