# Supplementary material for: Molecular dynamics provides new insights into the mechanism of calcium signal transduction and interdomain interactions in cardiac troponin
Source: FEBS Open Bio. 2021 Jun 9;11(7):1841–53. doi: 10.1002/2211-5463.13009 (PMC8255835; doi:10.1002/2211-5463.13009)
Supplement: Supplementary file 1 — Video S1. Ca2+ binding into TnC site II. Figure S1: Coordination of Ca2+ in Site II Figure S2: Ca2+ saturated state of troponin: N2‐OE12/N9‐OE12 interaction Figure S3: Straightening of TnI C‐terminal α‐helical extension Figure S4: The achieved coordination of Ca2+ after rebinding Figure S5: Escape route of the Ca2+ ion Figure S6: Extracting the TnI switch region from the TnC A/B hydrophobic pocket by using SMD [file FEB4-11-1841-s001.zip › feb413009-sup-0001-FigS1-6.pdf]

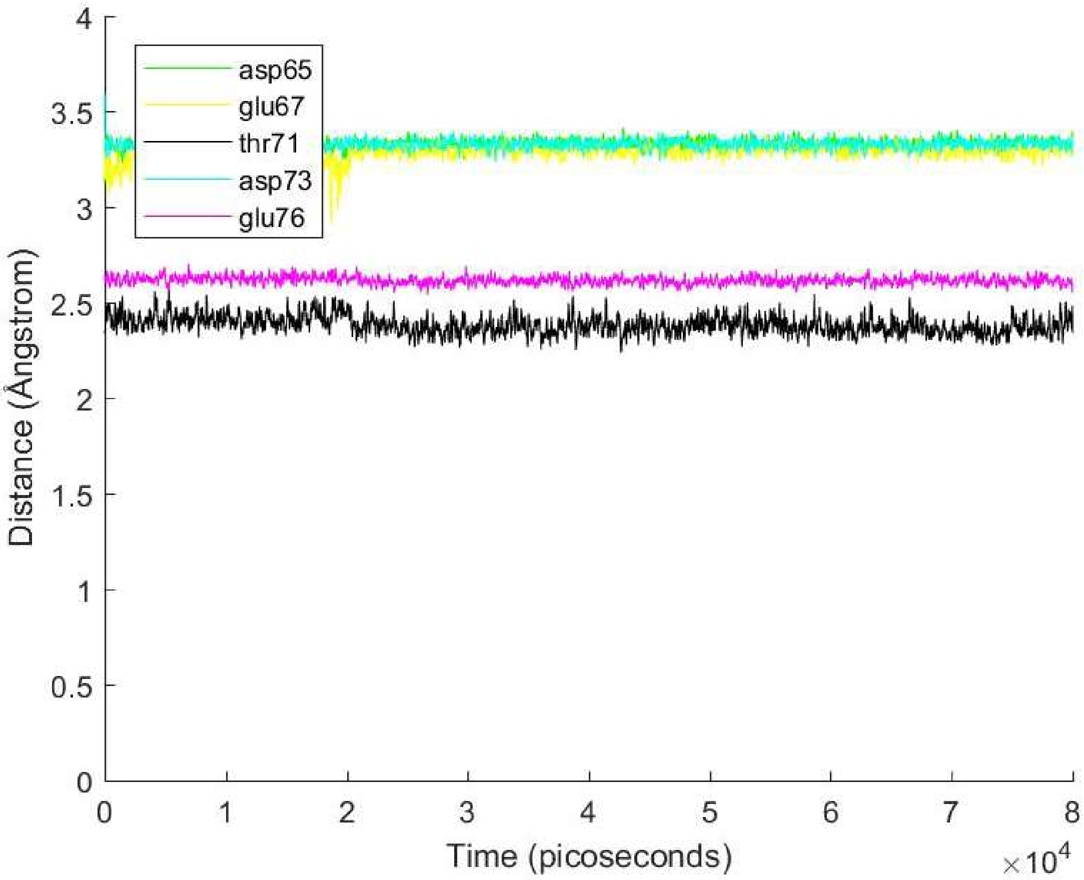

Figure S1: Coordination of Ca^{2+} in Site II

Distances between the Ca^{2+}ion and Asp65, Glu67, Thr71, Asp73, and Glu76 are shown for the duration of 80 ns of Ca^{2+} saturated state simulation of cardiac troponin. Separating distances are well-maintained between the ligands and the Ca^{2+} ion.

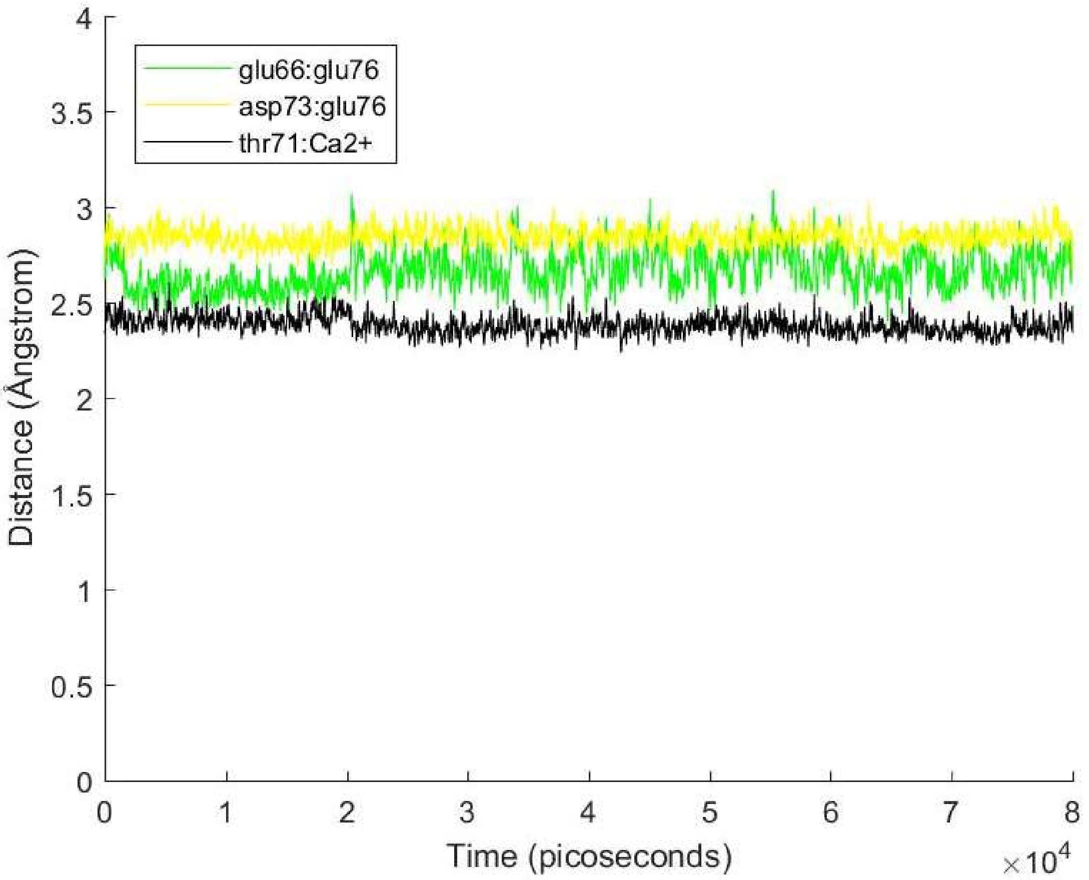

Figure S2: Ca^{2+} saturated state of Tn: N^2-OE^{12}/N^9-OE^{12} interaction

80 ns of Ca^{2+} saturated state simulation of cardiac troponin. Distance between 12/Glu76OE:2/Glu66HN (green), 12/Glu76OE:9/Asp73HN (yellow), and Ca^{2+} ion to residue Thr71O (black) shown to highlight Ca^{2+} bound state. The N^2-OE^{12}/N^9-OE^{12} interaction is well-maintained in the Ca^{2+} bound state.

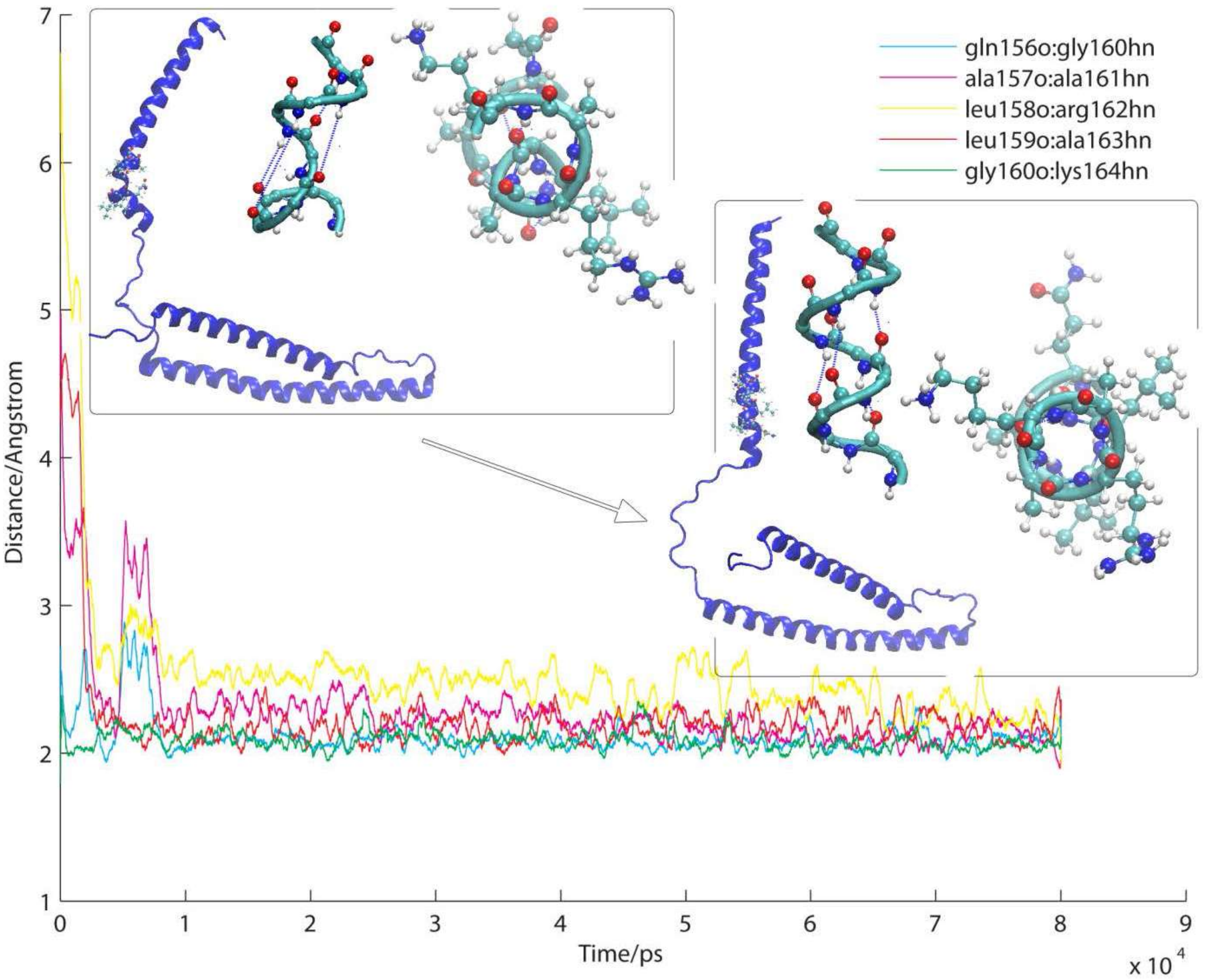

Figure S3: Straightening of TnI C-terminal α-helical extension

α-Helical contacts i to i+4 at the TnI bend hinge. Flanking contact are Gln156-Gly160 and Gly160-Lys164, which are well formed at the start, In-between contacts Ala157O-Ala161HN, Leu158O-Arg162HN, Leu159O-Ala163HN reform within ~10 ns of simulation time and the TnI C-terminal extension settles as a fully extended α-helix without a bend.

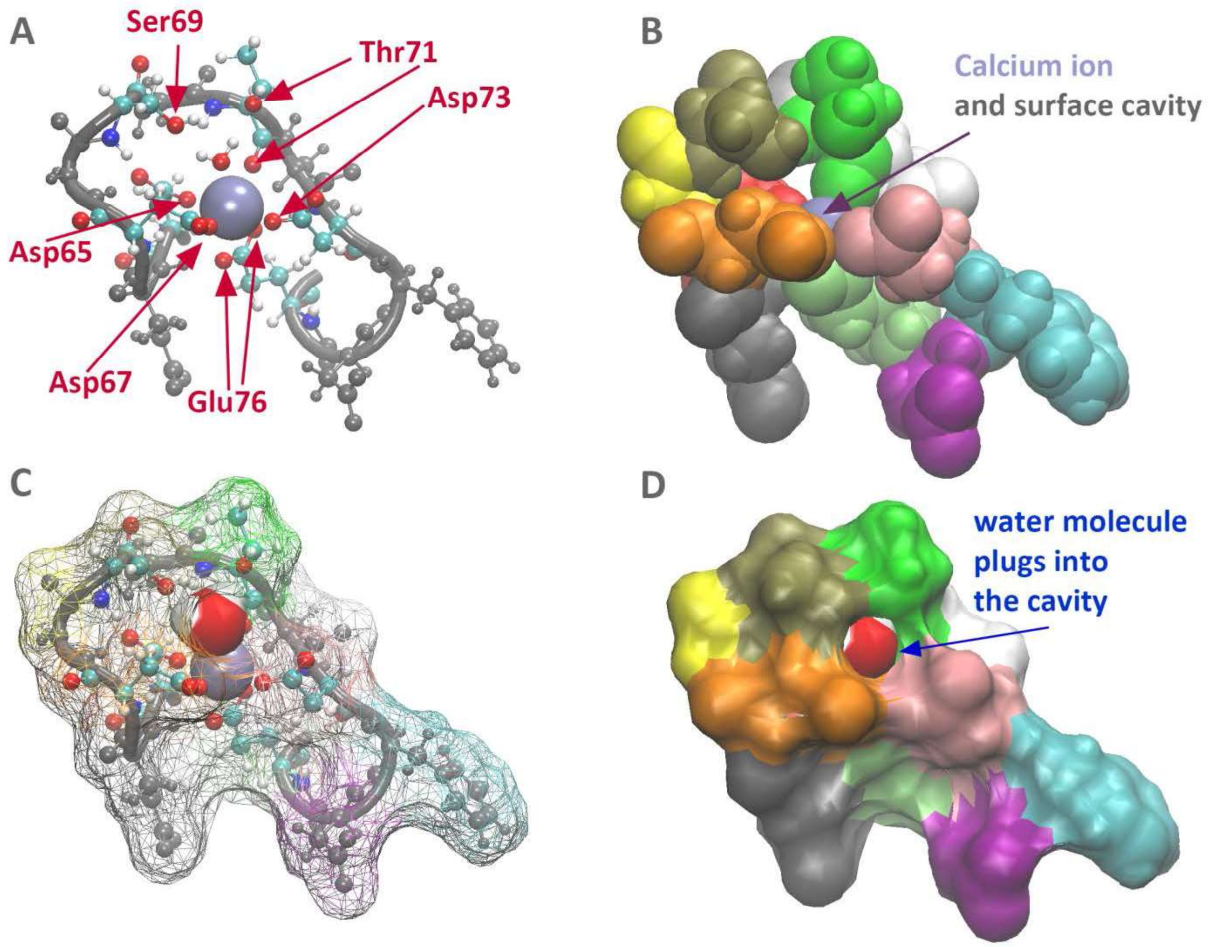

Figure S4: The achieved coordination of Ca^{2+} after rebinding

Panel A: Coordination of Ca^{2+}: Residue Asp65, mono-dentate via side chain oxygen; residue Asp67, mono-dentate via side chain oxygen, residue Ser69, mono-dentate via water and side chain oxygen, residue Thr71 twice, (first by side chain oxygen via water molecule and by Thr71 main chain oxygen); residue Asp73 mono-dentate via delta oxygen; residue Glu76 bi-dentate via delta oxygen. The side chain coordination of residues Ser69 and Thr71 is via a water molecule which plugs into the coordination cavity.

Panel B: Coordination atoms of Ca^{2+} leave a cavity (VDW and wireframe SASA probe 1.4Å representation of site II.

Panel C and **Panel D**: Water plugs in the outside cavity.

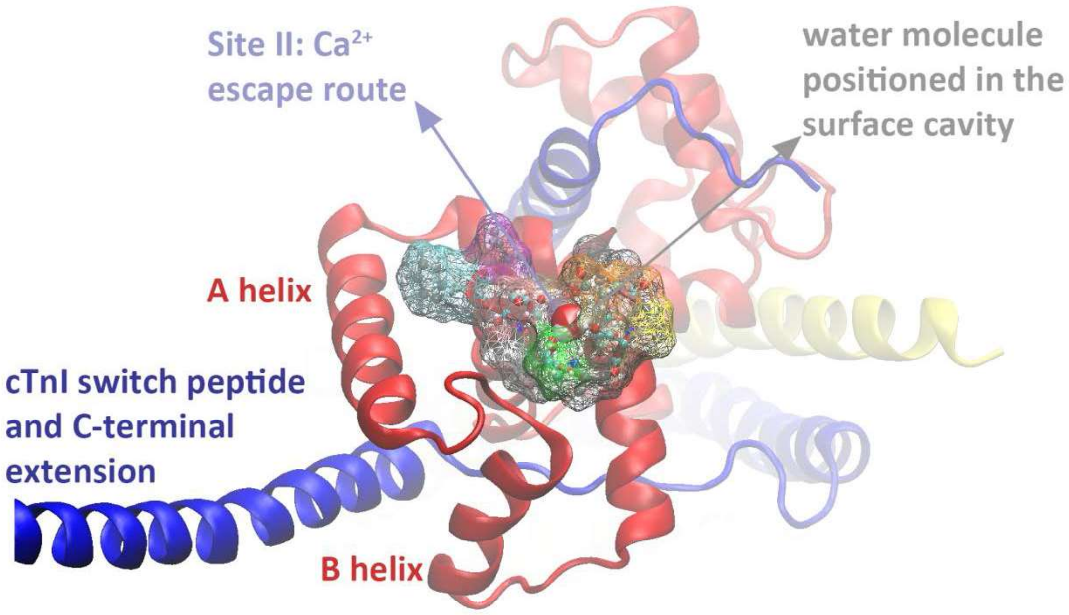

Figure S5: Escape route of Ca^{2+} ion

Escape route of Ca^{2+} via the coordination loop cavity (shown plugged-in by a water molecule). TnC shown in red, TnT shown in yellow and TnI shown in blue. Key structural features are labeled accordingly. The Ca^{2+} binding site is shown as wireframe probe 1.4 Å surface representation.

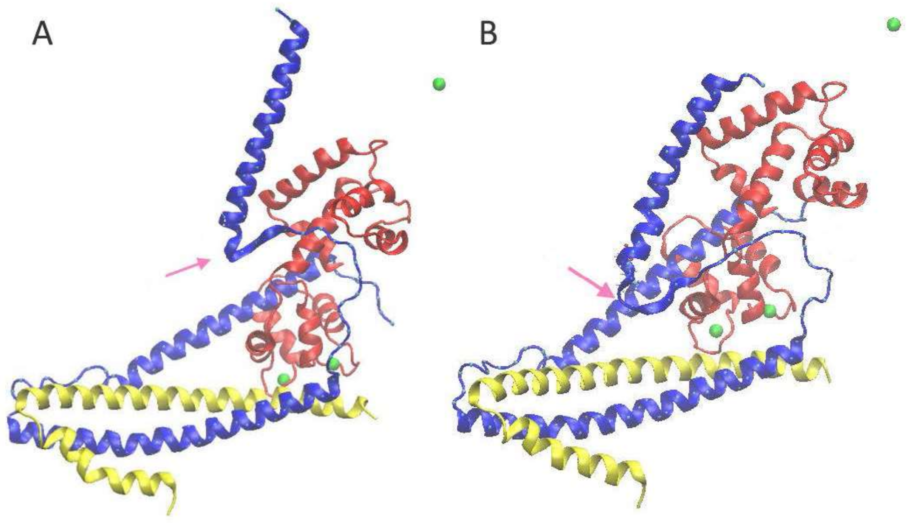

Figure S6: Extracting the TnI switch region from the TnC N-lobe hydrophobic pocket using SMD

Panel A: C-alpha atoms of the TnC N-lobe hydrophobic pocket are harmonically constrained. The TnI switch displacement from the TnC N-lobe hydrophobic pocket is achieved by applying steering force to the C-alpha atom of residue 156 (pink arrow). The point-of-pulling atom is translated ~20 Å.

Panel B: All atoms of TnC N-lobe hydrophobic pocket residues are harmonically constrained. The TnI switch displacement from the TnC N-lobe hydrophobic pocket is achieved by applying steering force to the C-alpha atom of residue 156 (pink arrow). The point-of-pulling atom is translated ~60 Å.
